# Single-cell RNA sequencing analysis of bone cancer pain model induced by Lewis lung cancer cells in male mice

**DOI:** 10.1080/07853890.2026.2636340

**Published:** 2026-02-27

**Authors:** Qin Zhou, Jiahui Chen, Hui Wang, Ruyi Jin, Halisa Paerhati, Xiuxiu Zhu, Gaochao Dong, Minhao Zhang, Feng Jiang

**Affiliations:** ^a^ Jiangsu Province Key Laboratory of Anesthesiology, Xuzhou Medical University, China; ^b^ Jiangsu Cancer Hospital & Jiangsu Institute of Cancer Research, The Affiliated Cancer Hospital of Nanjing Medical University, China; ^c^ Department of Anesthesiology, Eye & ENT Hospital of Fudan University, Shanghai, China

**Keywords:** Bone cancer pain, spinal cord, oligodendrocytes, microglia, single-cell RNA sequencing

## Abstract

**Background:**

Bone cancer pain (BCP) is one of the most severe complications faced by cancer patients, with complex physiological and pathological mechanisms and unclear molecular characteristics.

**Methods:**

The BCP model was established by inoculating Lewis lung cancer cells into the femur to induce hyperalgesia and spontaneous pain. Single-cell RNA sequencing technology was used to characterize the cell composition and molecular features of the L2-L4 spinal cord after BCP modelling.

**Results:**

Our research results identified a total of 10 cell types, namely excitatory neurons, inhibitory neurons, oligodendrocytes, oligodendrocyte precursor cells, Schwann cells, astrocytes, microglia, endothelial cells, fibroblasts, and pericytes. RNA sequencing analysis of the BCP model showed that the proportion of cells in the L2-L4 spinal cord changed significantly, with microglia increased by 45% and oligodendrocytes increased by 43%. Then, data were extracted from microglia, oligodendrocytes, blood-spinal cord barrier component cells (endothelial cells, pericytes, astrocytes), excitatory neurons, and inhibitory neurons, and differential genes were analysed and further enriched. The results suggest that the signalling pathways related to pain perception and transmission and promoting inflammation in the above cells have changed significantly. Finally, this study revealed the interaction between L2-L4 spinal cord cells in BCP.

**Conclusions:**

These data help to understand the molecular mechanism changes caused by BCP and contribute to the development of new treatment methods.

## 1. Introduction

Bone cancer pain (BCP) is a complex pain state caused by primary tumours or other metastatic tumours. In the early stage, it often presents as intermittent dull or distending pain, which then progresses to continuous or breakthrough pain as the disease advances. This type of pain has the characteristics of nociceptive, inflammatory and neuropathic pain, and is prone to causing hyperalgesia [1]. Effectively controlling bone cancer pain is not only an important aspect of comprehensive cancer treatment, but is also closely related to the quality of life and survival time of cancer patients.

The mechanism of BCP is complex and includes peripheral and central mechanisms. The peripheral mechanism of bone cancer pain mainly includes bone tissue destruction, local tumour microenvironment, and physiological and biochemical changes in the dorsal root ganglion; the central mechanism mainly involves the spinal cord, and physiological and biochemical changes at the spinal cord and brain levels [2,3]. Importantly, the spinal cord is the main processing centre for nociceptive signals. When tumour cells produce inflammatory mediators that acidify the microenvironment or damage nerve endings, the spinal cord is overstimulated, leading to an increase or prolongation of pain signals, which are then transmitted to the higher central nervous system through the ascending pathway [4]. Therefore, it is necessary to study the molecular landscape changes in the L2–L4 spinal cord segments in the BCP model to clarify the specific mechanism of bone cancer pain and provide new treatment methods and targets for bone cancer pain patients.

Single-cell RNA sequencing (scRNA-seq) technology, as a breakthrough biological analysis method, can analyze the heterogeneity characteristics of cell populations at the whole-genome level. This technology can not only reveal the temporal changes in the dynamic transcriptional regulatory network during development, but also precisely capture the molecular response characteristics that are difficult to distinguish by conventional population sequencing under external stimulation [5,6]. With the continuous optimization of high-throughput detection platforms, this technology is promoting breakthroughs in the field of life sciences by accelerating the discovery of new cell subtypes and the precise localization of disease-related targets [7]. Tang PC et al.’s research used single-cell sequencing technology to discover a neuron-like macrophage subpopulation related to cancer pain and clarified the potential mechanism of cancer pain [8]. However, there are currently no published studies using single-cell sequencing technology to elucidate the changes in the molecular characteristics of the corresponding spinal cord segments in the bone cancer pain model. Therefore, the application of scRNA-seq will broaden our understanding of the composition and developmental regulation of L2-L4 spinal cord cells in the bone cancer pain model. In this study, we used scRNA-seq technology to explore the molecular characteristics of the L2-L4 spinal cord in the BCP model and revealed the dynamic communication between spinal cord cells in this situation.

## 2. Methods

### 2.1. Animals

All experiments were conducted using male C57BL6/J mice weighing between 20 and 25 grams. The mice were randomly divided into sham operation group and BCP group (with 10 mice in each group). Mice in each group were housed in cages with a 12-hour light/dark cycle and had unrestricted access to food and water. The animal experiments were conducted under the approval of the Nanjing Medical University Institutional Animal Care and Use Committee (IACUC) with approval number 2509032. The study has adhered to ARRIVE guidelines.

### 2.2. BCP model

The method for establishing a BCP model involves implanting Lewis lung carcinoma (LLC-Luc) cell line expressing luciferase into the bone marrow cavity of the right femur of mice [9]. LLC-Luc cell line was cultured in high-glucose (4.5 g/L) DMEM medium supplemented with 1% (v/v) penicillin and streptomycin (Gibco, Thermo Fisher Scientific) and 10% (v/v) foetal bovine serum (Gibco, Thermo Fisher Scientific). The culture conditions were 37 °C and 5% CO2. Once the cells were confirmed to be in good growth condition, they were digested with 0.05% trypsin and suspended in phosphatebuffered saline (PBS) at a concentration of 2 × 10^7 cells/mL for model inoculation.

After anaesthetizing the mice with 3% isoflurane, they were placed in a lateral position, shaved with depilatory cream, and the surgical site was disinfected with iodophor. The right knee joint of the mice was bent, and the white patellar ligament was observed. A 30 G needle was passed around the patellar ligament and inserted from the proximal intercondylar fossa position, drilling into the femoral medullary cavity along the long axis of the femur. A micro syringe was used to slowly inject 5 μL (1 × 105 cells) of tumour cells into the femoral medullary cavity, and the needle was left in place for a moment before being withdrawn. To prevent the leakage of tumour cells into the cavity, 2 μL of gelatin sponge solution was injected into the cavity. The wound was disinfected with an alcohol cotton ball, and then sutured in sequence. The sham operation group was injected with boiled and denatured tumour cells (5 μL, 1 × 105 cells) using the same operation method.

To assess the growth of tumour load, *in vivo* fluorescence imaging was performed using the luciferase activity of tumour cells. D-fluorescein (MCE, product name: HY-12591B) was intraperitoneally administered 15 min in advance. Under isoflurane anaesthesia, fluorescence images were captured using the Borutentan EyeView 100 multimodal animal *in vivo* imaging system (excitation wavelength filter: 670 nanometres, emission wavelength: 745 nanometres). The intensity of the fluorescent area was analysed using the TrueQuant imaging software (PerkinElmer Company).

### 2.3. Behavioural testing

All behavioural tests were conducted between 9:30 am and 2:30 pm The testing environment was ensured to be consistent, controlled by the same experimenter, and the experimenter was kept unaware of the groupings of the experimental subjects. Before the behavioural tests, the mice needed to adapt to the testing conditions for about 20 min. The mechanical abnormal pain and thermal hyperalgesia tests were conducted one day before the cell injection (0 day) and on the 7th, 14th, and 21st days after the LLC-Luc vaccine inoculation.

### 2.4. Paw withdrawal mechanical threshold (PWT)

The mechanical abnormal pain condition of mice was determined by using the plantar pressure test (PWT). We used von Frey filaments (Stortin Company, Wood Dale, Illinois) for the behavioral test. The mice were placed in an 8 × 8 × 8 cm plastic compartment for 30 min, with a mesh steel floor at the bottom of the compartment. The von Frey filaments (with a bending force ranging from 0.02 grams to 2.0 grams) were vertically applied to the plantar surface of the right hind paw of the mice. If the mice showed foot tremors or violent withdrawal, it was considered a positive reaction. The initial bending force was 0.4 grams, and the stimulation interval was at least 15 s. The 50% foot withdrawal threshold was calculated using the ‘up-down’ method [10].

### 2.5. Paw withdrawal latency

Paw withdrawal latency (PWL) assessed thermal hyperalgesia utilizing a plantar thermal pain tool (IITC Shanghai Yuyan Scientific Instrument Co., LTD., China). In brief, mice were separately habituated to the plexiglass compartments placed on the elevated glass plate for 30 min prior to the trial. In the right rear paw of mice, the radiant thermal stimulator was inserted below the glass palate underneath the plantar surface. Three heat stimuli were delivered 5 min apart. When the rear paw is lifted, retracted or licked, the stimulator was switched off, and the timer was terminated automatically. PWL was described as the average time to the endpoint. A time limit of 20 s was specified to prevent tissue harm.

### 2.6. H&E staining

After the behaviour test at POD21, the fresh mouse femoral tissues (*n* = 6 per group), were placed in 10% formalin. Femurs from the model group and sham-operated group were collected, fixed in 4% paraformaldehyde solution for 24 h, decalcified in EDTA decalcification solution for 10 d, dehydrated in a graded ethanol series, embedded in paraffin, and sectioned (4 μm thick) [11]. Haematoxylin–eosin staining was performed, and pathological damage to the tumour-bearing femurs was observed under an upright optical microscope.

### 2.7. micro-CT

Micro-CT imaging was performed on the BCP group and the sham group of mice at 21 days (Micro-CT provided by Pingsheng Medical Technology Company; Version: VNC-102). After the imaging process, data is collected using Cruiser and the images are reconstructed using Recon. Quantification of micro-CT data was calculated for distal femurs of Sham and BCP mice. Parameters included BS/BV, Conn.D, and Tb.N within a region of 1 mm proximal to the distal growth plate.

### 2.8. scRNA-seq of mouse L2-L4 spinal cord

Single-cell nuclear suspension was added to the 10x Chromium chip according to the instructions for the 10X Genomics Chromium Single-Cell 3′ kit (V3), with the expectation of capturing 8,000 cells. cDNA amplification and library construction were performed according to standard protocols. Libraries were sequenced by LC-Bio Technology (Hangzhou, China) on an Illumina NovaSeq 6000 sequencing system (double-end sequencing, 150 bp) at a minimum depth of 20,000 reads per cell.

### 2.9. Bioinformatics processing

Bioinformatic analysis of scRNA-seq were conducted by LC-bio (Hangzhou, China).

First, the matrices of the two samples were obtained according to the 10 Genomics Cellranger software (version 3.1, 10 × Genomics) protocol. Then, the libraries were sequenced by LC-Bio Technology (Hangzhou, China) on the Illumina NovaSeq 6000 sequencing system (paired-end sequencing, 150 bp). Results from Illumina sequencing offline were converted to FASTQ format using bcl2fastq software (version 5.0.1). The scRNA-seq sequencing data were compared to reference genome using CellRanger software, and cellular and individual cellular 3′ end transcripts were identified and counted in the sequenced samples. (https://support.10xgenomics.com/single-cell-gene expression/software/pipelines/latest/what-is cell-ranger, version 7.0.0). The output CellRanger expression profile matrix was loaded into Seurat (version 4.1.0) for filtering of low quality cells from scRNA-seq data, and the filtered data was downscaled and clustered. Filtering low cell quality thresholds: number of genes expressed per cell >500, mitochondrial genes expressed in <25% of cells. Cells were projected into 2D space using t-SNE or UMAP. These steps include: 1. calculating gene expression values using the LogNormalize method of Seurat’s “NormalizeData” function; 2. performing principal component analysis (PCA) using the normalized expression values, using the top 20 PCs for clustering, and Findcluster analysis; 3. analyze the marker genes of each cluster based on Findallmarker, and the marker genes were selected based on the following criteria: expressed in more than 10% of cells in each cluster, P value ≤ 0.01, gene expression ploidy logFC ≥ 0.26; Hypergeometric testing was used to perform GO and KEGG enrichment analysis on the differential genes of each cluster obtained from Findallmarker analysis relative to other clusters. In human and mouse species, singleR database, scCATCH database and our self-developed LC-Marker were used to identify each cluster cell type, and some cells were re-clustered based on the identification results of SingleR database to find marker genes, cell communication analysis and Pseudotime analysis. GO enrichment were analyzed at http://geneontology.org(version 2021.4). KEGG enrichment were analyzed at http://www.kegg.jp/kegg (version 2021.4).

### 2.10. Pseudotime analysis

The Monocle 2 R package (https://www.omicstudio.cn/cell) was used to perform pseudotime analysis. The order cells function was used to arrange and display the cells along the quasi-chronological path.

### 2.11. Intercellular interaction network analysis

Cell–cell communication was computed *via* ligand–receptor interactions. The cell–cell communication network was constructed and visualized using Cell Chat software (https://www.omicstudio.cn/cell).

### 2.12. Statistical analysis

Values are expressed as mean ± SEM. Data from the two groups underwent statistical analysis using two-tailed unpaired Student’s *t*-tests, assuming equal variance. GraphPad Prism 10.1.2 (GraphPad Software, San Diego, CA, USA) was used for statistical analyses, with *p* < 0.05 considered statistically significant.

## 3. Result

### 3.1. Establishment of bone cancer pain model

**Figure 1.**
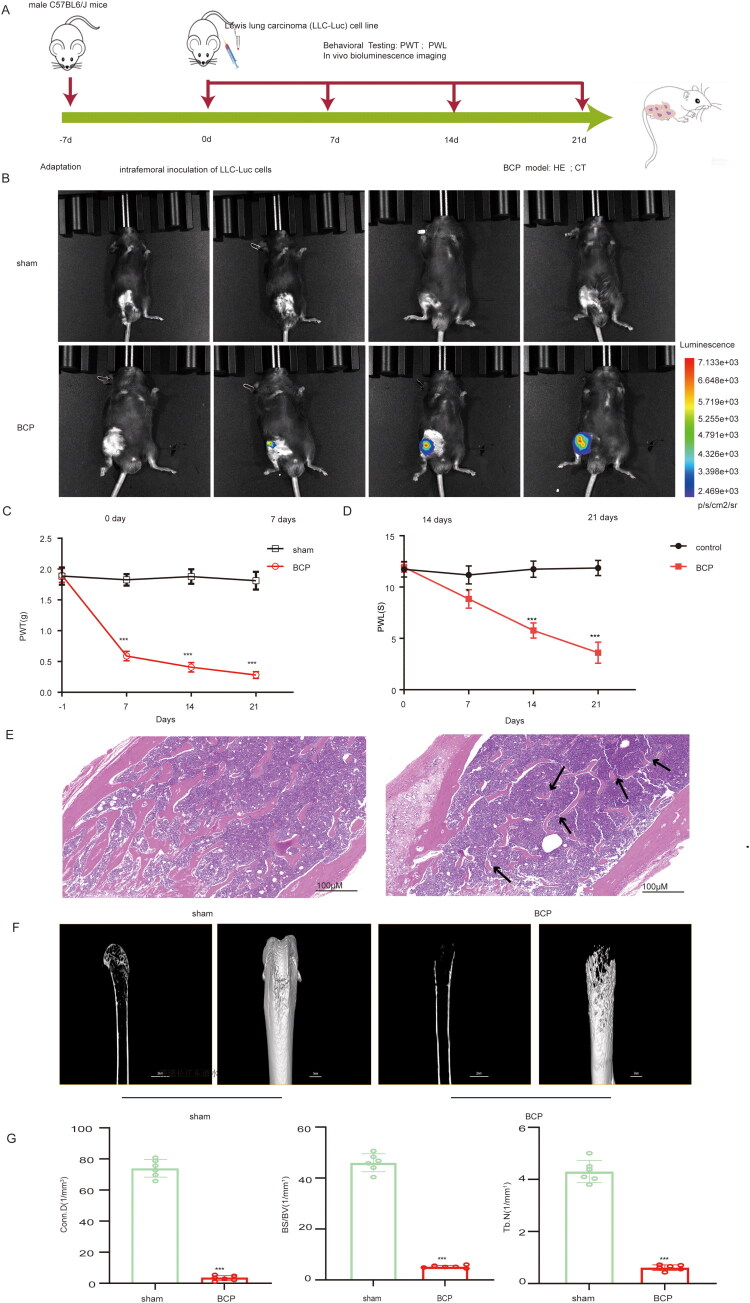
Establishment of bone cancer pain model (A) Flow chart of BCP moulding. (B) *In vivo* bioluminescence imaging showed that after 7 days of introfemoral injection of LLC-Luc cells, Tumour cell growth was observed in the femur. (C-D) Examination of mechanical and thermal pain behaviour using von Frey test to determine PWT and Hargreaves tests to determine heat hyperalgesia. BCP mice showed increased mechanical or thermal hypersensitivity on POD7 compared to sham operated mice. (E) H&E staining showed bone structure destruction and marrow cavity infiltration in the BCP mice compared with the sham operation mice that received heat-killed LLC-Luc cells. (F) CT results indicated significant destruction of the femur structure and bone mass in the BCP mice compared with the sham-operation mice. (G) The analysis of Conn.D, BS/VV and Tb.N. Data are expressed as the mean ± SEM and analysed using two-way repeated-measures ANOVA with post hoc Bonferroni test; *p < 0.05, ***p < 0.001; *n* = 6 per group, scale: 100 μm.

The specific modelling process of BCP was shown in Figure 1(A): LLC-Luc cells expressing luciferase were inoculated into femur of male C57BL/6J mice to establish the BCP model. First, to demonstrate tumour development in the femur, luciferase signals were detected by bioluminescence *in vivo* from POD7 to POD21 (Figure 1(B)). Secondly, mechanical or thermal plantar stimulation was performed on sham operation and BCP mice, respectively. The results showed that, compared with the sham-operated mice, the BCP mice had intense mechanical pain and thermal hyperalgesia observed from POD7 to POD21 (Figure 1(C–D)). Thirdly, HE staining and CT imaging of femur were performed in POD21, respectively, demonstrating that the mouse femur showed xenotypic cell infiltration into the bone marrow cavity and the destruction of femur bone by tumour (Figure 1(E–F)). Finally, BCP mice exhibited marked bone destruction, as revealed by sharp decreases in Conn.D, BS/VV and Tb.N (Figure 1(G)). In summary, the results validate our mouse BCP model.

### 3.2. scRNA-seq of L2-4 spinal cord with BCP

**Figure 2.**
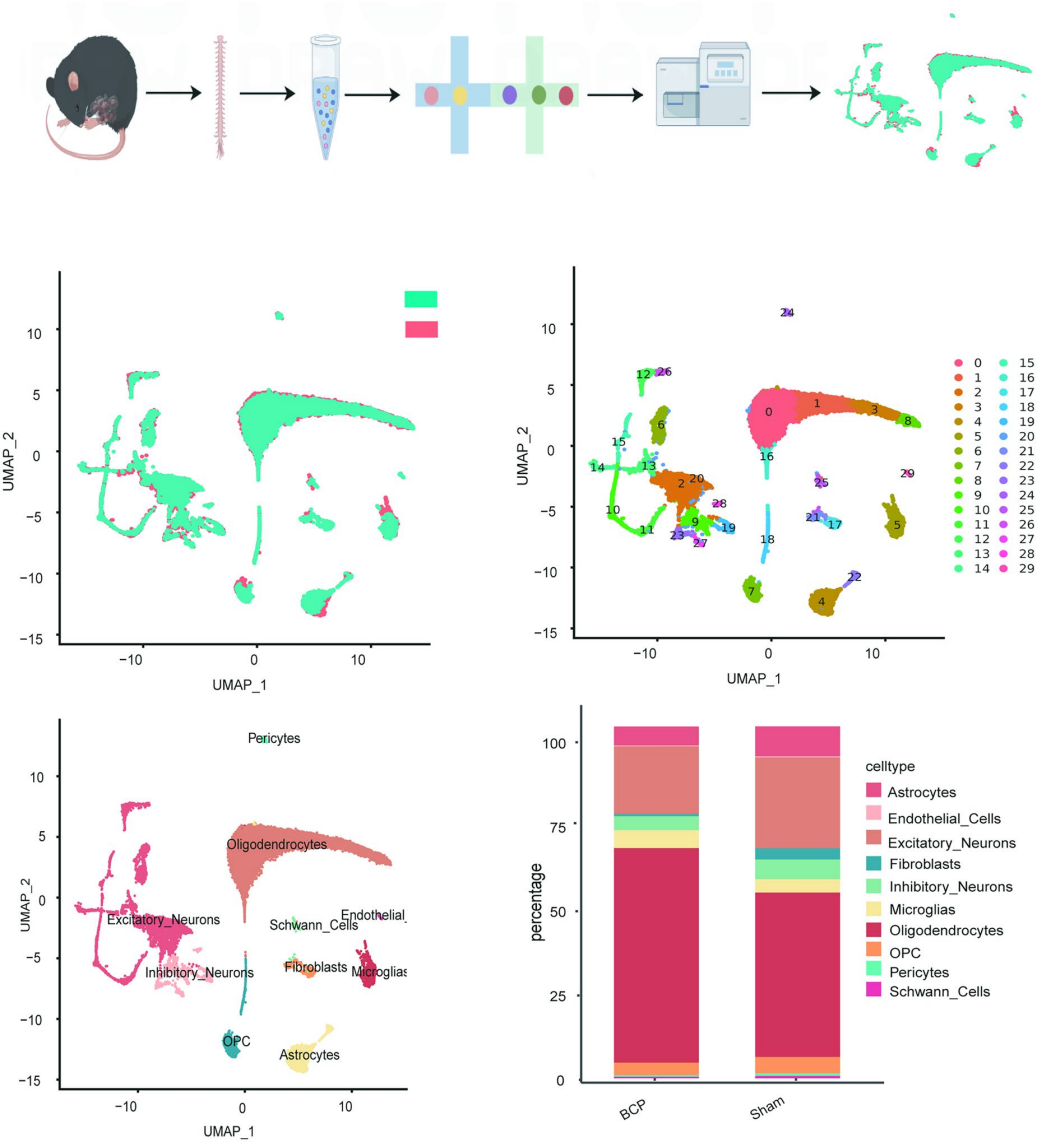
Identification of cell composition in mouse L2-L4 spinal cord tissue using single-cell RNA sequencing (A) Workflow diagram of the single-cell sequencing experiment. (B) UMAP visualization shows 30 cell clusters identified from a total of 24,993 single-cell transcriptomes from the control group and the BCP group. (C) Ratio of cells between the control group and the BCP group.

The flowchart of our entire experiment is shown in Figure 2(A). After successfully establishing the mouse BCP model, we aimed to determine the precise molecular characteristics of the cells at the L2-L4 spinal cord level 21 days after BCP. We conducted scRNA-seq experiments on the L2-L4 spinal cord tissues from sham and BCP mice (Figure 2(B)). After eliminating low-quality cells, a total of 24,993 cells were obtained (Control: 11,744, BCP: 13,249). On average, each cell contained 1,447 genes, with a total of 16,729 genes. After integration, dimension reduction, and clustering, the cells were divided into 29 clusters. Based on specific marker genes, these cells were classified into 10 cell types (Figure 2(B)). Through uniform manifold approximation and projection (UMAP), the cell types were identified as excitatory neurons, inhibitory neurons, oligodendrocytes, OPCs, Schwann cells, astrocytes, microglia, endothelial cells, fibroblasts, and pericytes. The proportion analysis of spinal cord tissue cells showed that the proportions of excitatory neurons, astrocytes, and fibroblasts decreased in the BCP group, while the proportions of oligodendrocytes and microglia significantly increased (Figure 2(C) and Table 1). These results suggest that BCP-induced spinal cord injury may affect neural function and pain transmission by altering cell components.

**Table 1.** The cell number of each cell type is shown by single-cell sequencing.

Cell type	Group	count
Microglia	Sham	400
Oligodendrocytes	BCP	580
	Sham	4888
Astrocytes	BCP	6994
	Sham	893
Excitatory Neurons	BCP	620
	Sham	2700
Inhibitory Neurons	BCP	2205
	Sham	585
Endothelial Cells	BCP	457
	Sham	33
Pericytes	BCP	29
	Sham	73
Schwann Cells	BCP	52
	Sham	70
Fibroblasts	BCP	53
	Sham	334
OPC	BCP	75
	Sham	491
	BCP	405

### 3.3. Analysis of oligodendrocyte gene expression patterns in BCP

**Figure 3.**
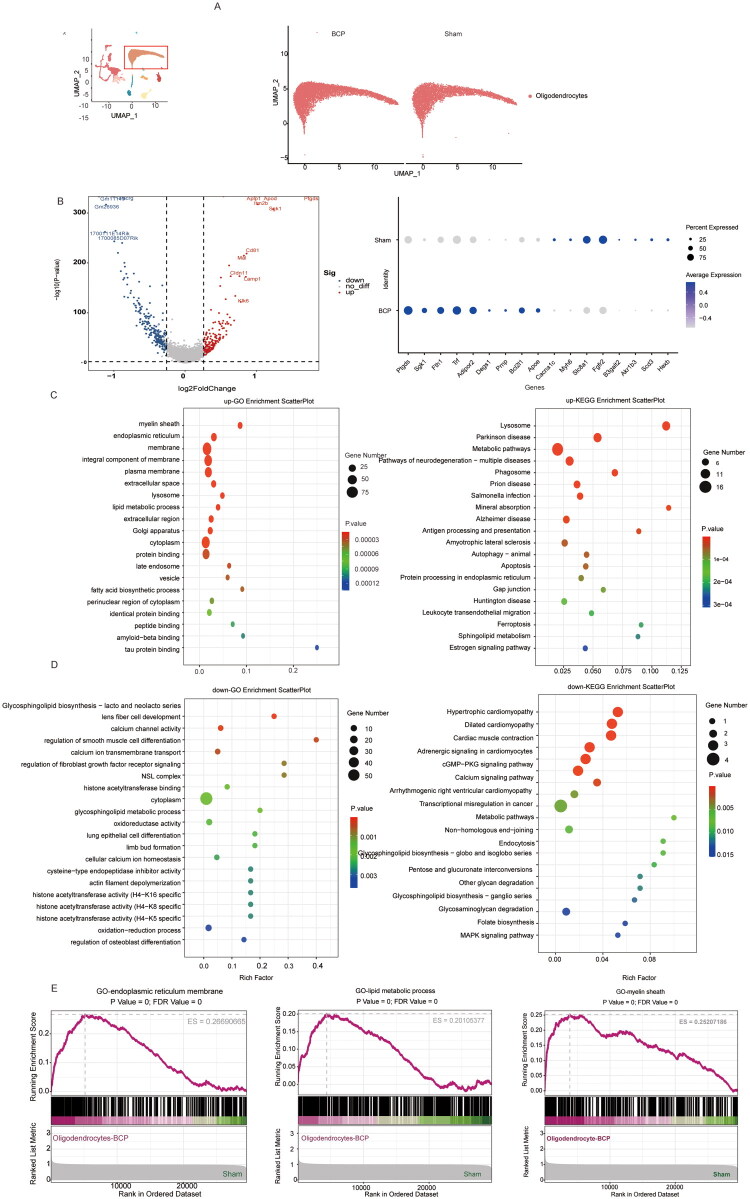
The characteristics of the gene expression profile of oligodendrocytes after BCP modelling. (A) UMAP visualization of oligodendrocytes in the sham and BCP groups. (B) Volcano plot of differentially expressed genes in oligodendrocytes between the control group and the BCP group, as well as dot plots showing the expression levels and frequencies of some of the differentially expressed genes. (C) GO analysis and KEGG analysis of genes whose expression was upregulated after BCP injury. (D) GO analysis and KEGG analysis of genes whose expression was downregulated after BCP injury. (E) GSEA of GO after BCP injury. *p* < 0.05 was significantly enriched.

In previous studies on BCP, the scientific community’s focus was mainly on the activation of microglia and astrocytes and their roles in pain regulation. However, relatively less attention was paid to another important type of glial cell in the spinal cord tissue – oligodendrocytes. The single-cell RNA sequencing results of this study revealed a phenomenon worthy of in-depth exploration: compared with the sham surgery group, the proportion of oligodendrocytes in the BCP model group showed a significant upward trend (Figure 2(C)). This finding prompted us to conduct a more in-depth exploration of the potential mechanism of oligodendrocytes in the occurrence and development of bone cancer pain. Next, we clarified the changes in the gene expression profile of oligodendrocytes after modelling. By comparing the different genes of oligodendrocytes in the sham group and the BCP group (Figure 3(A)); Based on the identified differentially expressed genes, corresponding volcano plots were obtained, and the dot plots indicated that genes such as Ptgds, Sgk1, scd2, Fth1, Trf, Adipor2, Degs1, Prnp, Bcl2l1, and Apoe were significantly upregulated, while genes such as Cacna1c, Slc8a1, Fgfr2, Slc8a1, B3galt2, Akr1b3, Scd3, and Hexb were significantly downregulated (*p* < 0.05, Figure 3(B)). Further Gene Ontology (GO) and Kyoto Encyclopaedia of Genes and Genomes (KEGG) analyses were conducted on the upregulated and downregulated differentially expressed genes (Figure 3(C–D)). The results showed that oligodendrocytes were significantly enriched in the aspects of myelin formation and maintenance, suggesting abnormal expression of genes involved in maintaining myelin function may lead to myelin dysfunction. In terms of cellular stress responses, the enrichment of endoplasmic reticulum stress and lysosomal function indicated that oligodendrocytes might be in a persistent stress state. Particularly noteworthy was the activation of ferroptosis-related pathways, which might represent a new mechanism of cell damage. Additionally, the disorder of calcium signalling pathways not only could affect the functions of oligodendrocytes themselves, but also might participate in the conduction of pain signals by altering the interaction between neurons and glial cells. The corresponding Gene Set Enrichment Analysis (GSEA) analysis also indicated that under the BCP model, oligodendrocytes had abnormalities in endoplasmic reticulum function, lipid metabolism, and myelin sheath (Figure 3(E)). These findings collectively present a dynamic picture of the changes in oligodendrocytes under bone cancer pain conditions: Under pathological stimulation, oligodendrocytes not only increase in number, but also undergo significant reprogramming of their gene expression profiles, mainly involving multiple key biological processes such as metabolism, stress response, and myelin maintenance. These changes may lead to the disruption of myelin structure and abnormal nerve conduction function, and may further participate in the central transmission of pain signals by altering the activity of neurons.

### 3.4. Analysis of microglial cell gene expression profiles after BCP model establishment

**Figure 4.**
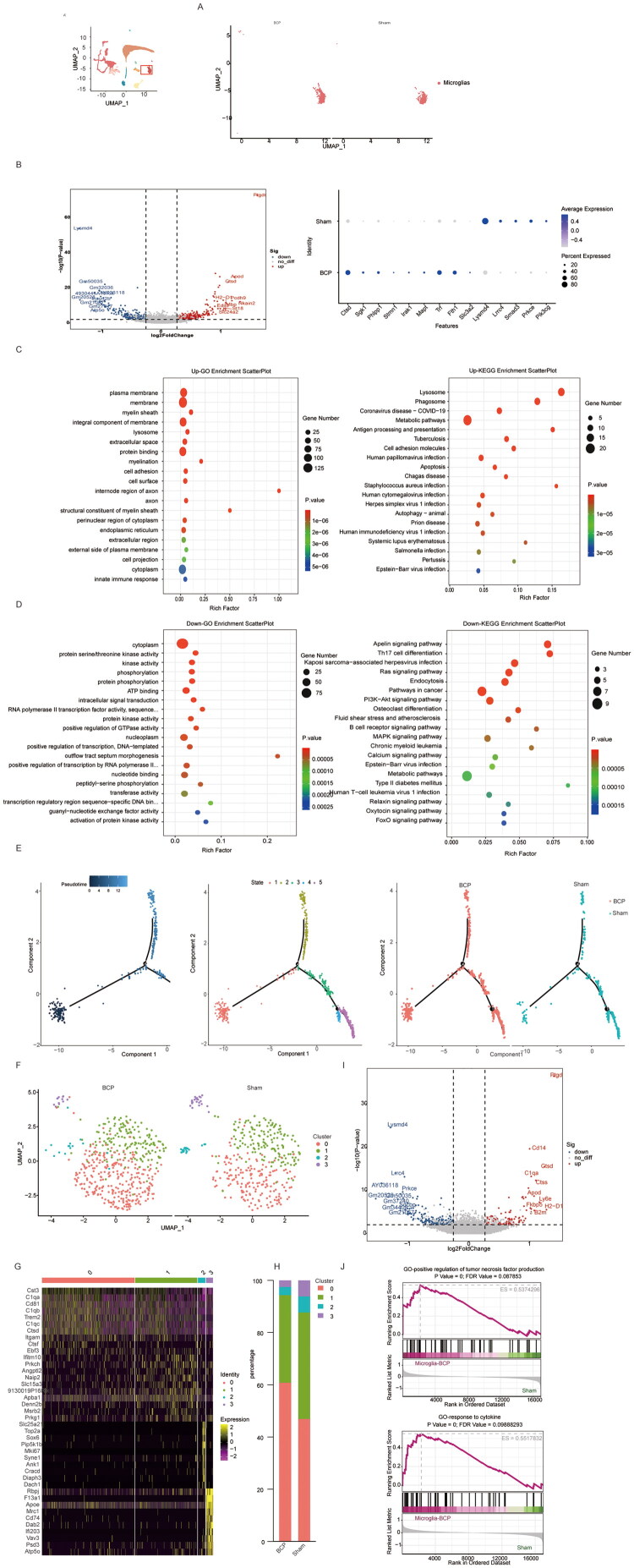
The characteristics of the gene expression profile of microglia after BCP modelling. (A) UMAP visualization of microglia in the sham and BCP groups. (B) Volcano plot of differentially expressed genes in microglia between the control group and the BCP group, as well as dot plots showing the expression levels and frequencies of some of the differentially expressed genes. (C) GO analysis and KEGG analysis of genes whose expression was upregulated after BCP injury. (D) GO analysis and KEGG analysis of genes whose expression was downregulated after BCP injury. (E). Pseudo-time analysis of the trajectory of microglia. Pseudo-time values are colour-grade from dark blue (undifferentiated) to dark red (differentiated) cellular states. (F) UMAP visualization of microglia after BCP. (G) Heatmap of specific gene markers used to annotate microglia subtypes. (H) Percentage of microglia between the Sham and BCP groups. (I) Volcano plot of differentially expressed genes in microglia cluster 0 between the Sham and BCP groups. (J) GSEA analysis on the differentially expressed genes in microglia cluster 0 between the Sham and BCP groups. *p* < 0.05 was significantly enriched.

Microglia are the main immune cells in the central nervous system and have always been one of the target cells in bone cancer pain research. Most studies have shown that microglia mainly promote the occurrence and development of bone cancer pain through mechanisms such as neuroinflammation, synaptic remodelling, and intercellular interactions. Our single-cell sequencing results also suggest that the proportion of microglia increases after BCP. Next, we will further analyze the changes in the gene expression profile of microglia after BCP. Many genes related to this mechanism have shown significant changes in expression (Figure 4(A)). Compared with the control group, in the BCP group, genes such as Ctsd, Sgk1, Magi2, Phlpp1, TSsc1, Stmn1, Irak1, Mapt, Ttaf3, Trf, Fth1, Slc3a2 are upregulated, while genes such as Lysmd4, Lrrc4, Smad3, Prkce, Pik3cg are downregulated (*p* < 0.05, Figure 4(B)). Upregulated genes such as Ctsd, Sgk1, etc., are closely related to lysosomal metabolism, PI3K-Akt signalling pathway, and ferroptosis regulation [12,13]. The abnormal expression of these genes may exacerbate the inflammatory response and metabolic disorder of microglia. On the other hand, downregulated genes such as Lysmd4, Lrrc4, Smad3, Prkce, Pik3cg, etc., are known to be related to the regulation of neural inflammation and the maintenance of synaptic homeostasis, and their reduced expression may lead to the uncontrolled inflammatory response and abnormal synaptic function [14,15].

GO analysis and KEGG analysis were performed separately on the upregulated and downregulated genes (Figure 4(C–D)). The results showed that microglia in BCP exhibited obvious characteristics of neuroinflammation and immune response, including innate immune response, autophagy, and abnormal activation or inhibition of several important signalling pathways. For example, the PI3K-Akt/MAPK signalling pathway is frequently involved in the regulation of inflammatory response, while the abnormal Apelin/FoxO signalling pathway may affect cell survival and function. In addition, the expression changes of ferroptosis-related genes suggest that microglia may participate in pain regulation through this mechanism. These data suggest that they may initiate a series of downstream inflammatory responses during BCP. Monocle pseudo-time analysis showed the state transmission of microglia cells in the BCP model (Figure4E). We characterized the subpopulations of microglia based on cell heterogeneity. We reclustered the microglia and obtained four clusters (Figure 4(F)). Among them, cluster 0 exhibited the most distinctive expression profile, and its proportion significantly increased after BCP (Figure 4(G–H)). Therefore, we further analyzed cluster 0. We conducted a differential gene expression analysis on cluster 0 (Figure 4(I)). Interestingly, we found that genes related to immune and inflammatory responses (such as Cd14) were expressed more abundantly (*p* < 0.05). At the same time, we performed GSEA analysis on the differentially expressed genes, and the results suggested that after BCP modelling, cluster 0 showed a significant positive enrichment in the positive regulation of tumour necrosis factor production and cytokine response pathways, indicating that cluster 0 is a highly activated microglial cell subset with a clear pro-inflammatory phenotype in the BCP model (Figure 4(J)). This subset may play a key role as a hub connecting tumour pathology, neuroimmune inflammation, and pain perception. This provides important cellular and molecular basis for understanding the molecular mechanism of BCP and developing targeted immunoregulatory analgesic strategies.

### 3.5. The characteristic changes in molecular markers of endothelial cells, pericytes and astrocytes induced by BCP

The blood-spinal cord barrier (BSCB), as an important protective structure of the central nervous system, is composed of endothelial cells, pericytes, astrocyte end feet, and basement membranes. Its function is similar to that of the blood–brain barrier and is crucial for maintaining the homeostasis of the spinal cord microenvironment [16]. However, in the BCP model, the integrity of the BSCB is significantly disrupted, leading to impaired barrier function and increased permeability, which in turn promotes the occurrence and development of pain [17].

**Figure 5.**
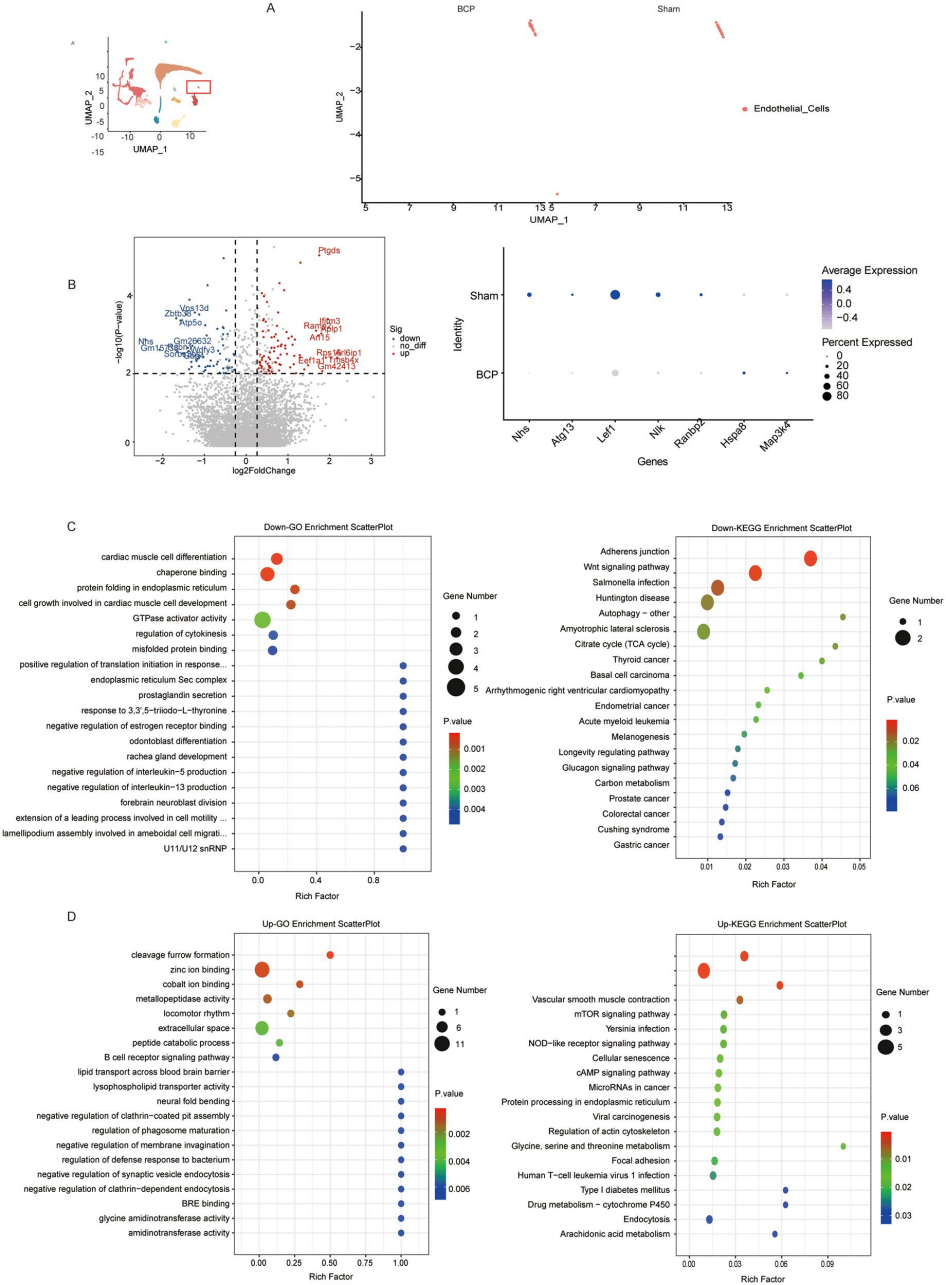
The characteristic changes in molecular markers of endothelial cells by BCP. (A) UMAP visualization of endothelial cells in the sham and BCP groups. (B) Volcano plot of differentially expressed genes in endothelial cells between the control group and the BCP group, as well as dot plots showing the expression levels and frequencies of some of the differentially expressed genes. (C) GO analysis and KEGG analysis of genes whose expression was downregulated after BCP injury. (D) GO analysis and KEGG analysis of genes whose expression was upregulated after BCP injury. *p* < 0.05 was significantly enriched.

Single-cell data analysis suggests that in the BCP model, the gene expression of endothelial cells undergoes significant changes (Figure 5(A)). Genes such as Nhs, Atg13, Lef1, Nlk, and Ranbp2 are downregulated, while Hspa8 and Map3k4 are upregulated (*p* < 0.05; Figure 5(B)). Further GO and KEGG analysis of the differentially downregulated genes indicates that the function of endothelial cells is significantly disrupted in the BCP model (Figure 5(C)). For instance, the downregulation of Lef1 can inhibit the Wnt pathway, suppress the expression of tight junction proteins, and cause BSCB disruption and increased permeability; the downregulation of Atg13 and Nhs can damage the integrity of endothelial cells and increase vascular permeability [18]. GO and KEGG analysis of the upregulated genes reveals that endothelial cells also exhibit activated inflammatory and immune response processes in the BCP model (Figure 5D). For example, abnormalities in the m-TOR signalling pathway and NOD-like receptor signalling pathway. In addition, the enrichment changes in extracellular space and lipid transport across the blood-brain barrier also suggest that the integrity of the blood–brain barrier maintained by endothelial cells is impaired.

**Figure 6.**
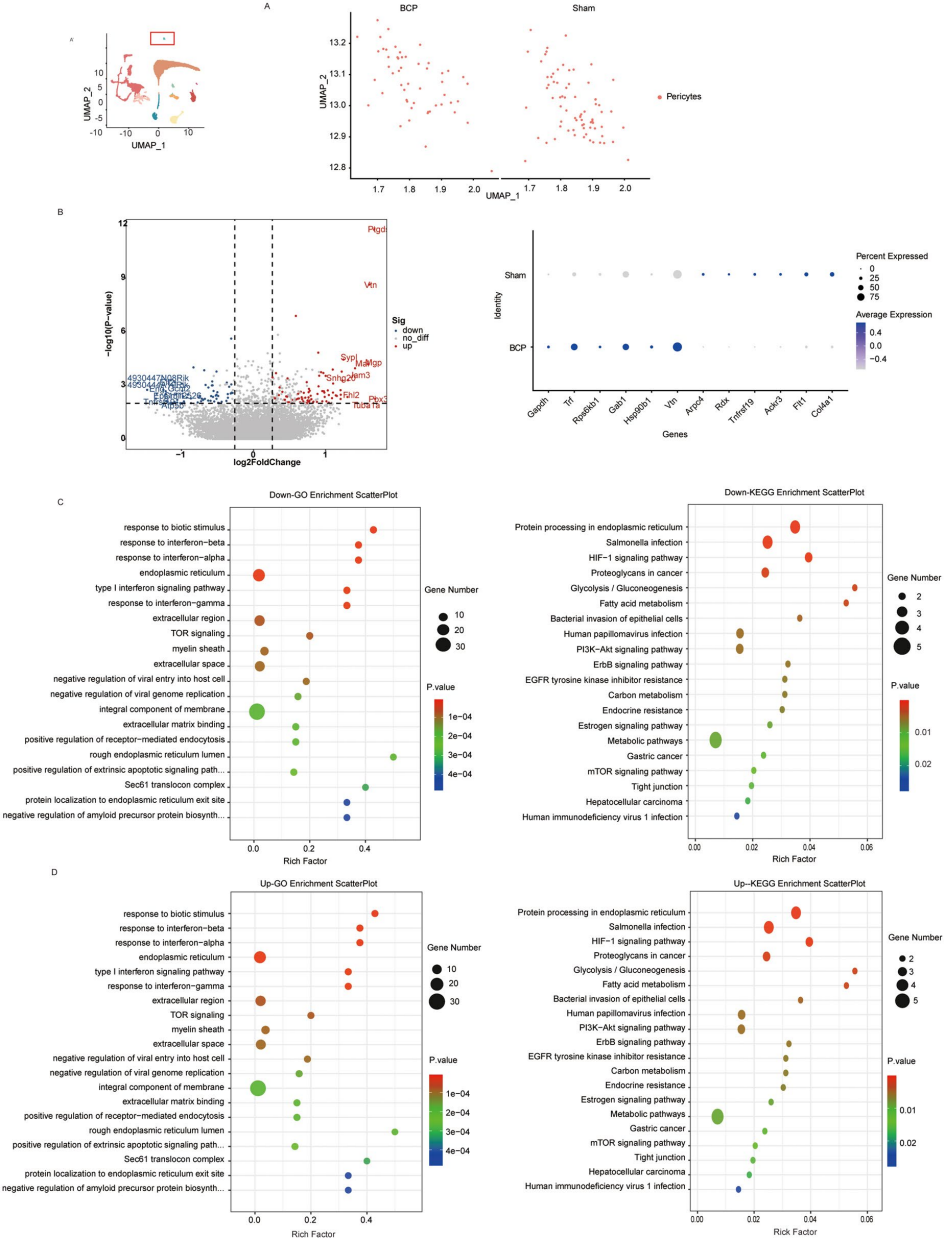
The characteristic changes in molecular markers of pericytes by BCP. (A) UMAP visualization of pericytes in the sham and BCP groups. (B) Volcano plot of differentially expressed genes in pericytes between the control group and the BCP group, as well as dot plots showing the expression levels and frequencies of some of the differentially expressed genes. (C) GO analysis and KEGG analysis of genes whose expression was downregulated after BCP injury. (D) GO analysis and KEGG analysis of genes whose expression was upregulated after BCP injury. *p* < 0.05 was significantly enriched.

After comparing the different genes in pericytes between the control group and the BCP group (Figure 6(A)), we found that Gapdh, Trf, Rps6kb1, Gab1, Rps6kb1, Hsp90b1, Vtn genes were upregulated, while Arpc4, Rdx, Tnfrsf19, Ackr3, Flt1, Col4a1 genes were downregulated (*p* < 0.05, Figure 6(B)). Further analysis of the GO and KEGG of the differentially downregulated and upregulated genes showed that in the BCP model, pericytes showed significant impairment in maintaining vascular integrity and permeability functions (Figure 6(C–D)). For instance, the downregulation of the TGF-β receptor signalling pathway led to vascular stability imbalance between pericytes and endothelial cells; Tight junction/Focal adhesion/ECM–receptor interaction indicated a downregulation in the role of pericytes in maintaining vascular barrier integrity. The activation of the HIF-1 signalling pathway led to an increase in vascular permeability. Additionally, pericytes also caused imbalances in immune/inflammatory responses. For example, the PI3K-Akt signalling pathway and type i interferon signalling pathways were imbalanced.

**Figure 7.**
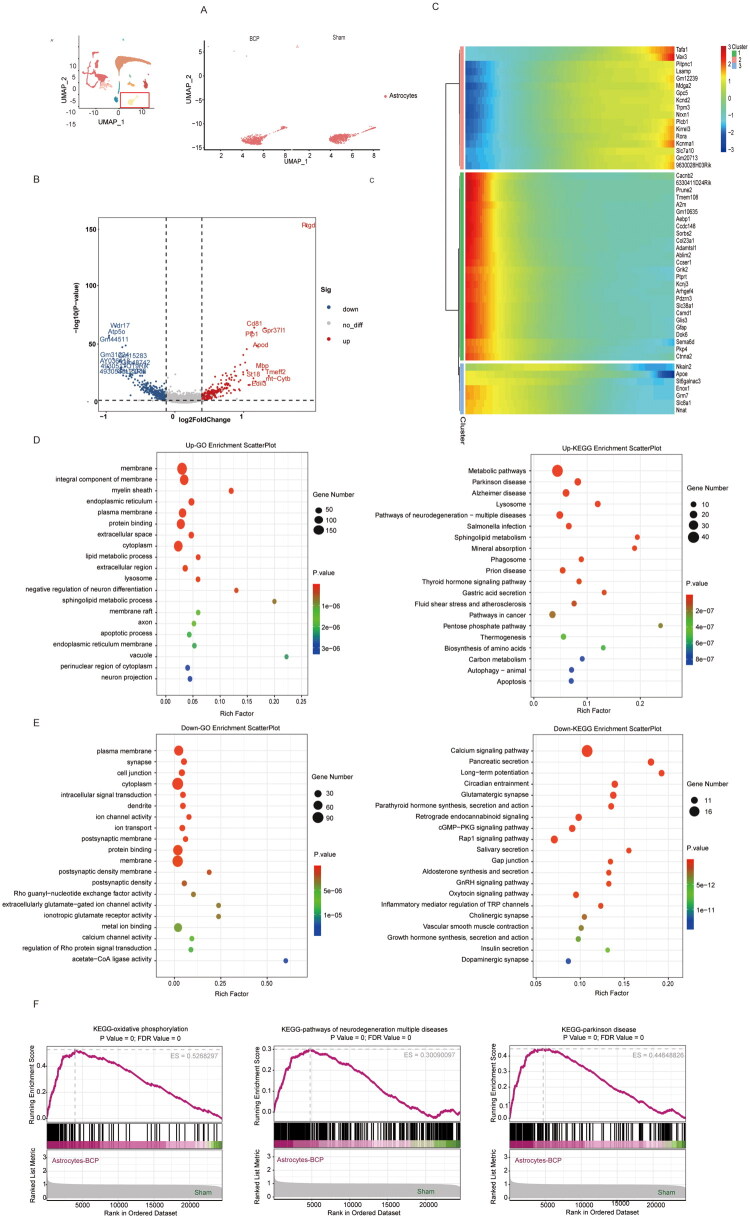
The characteristic changes in molecular markers of astrocytes by BCP. (A) UMAP visualization of astrocytes in the sham and BCP groups. (B) Volcano plot of differentially expressed genes in pericytes between the control group and the BCP group. (C) Hierarchical clustering of gene expression after BCP injury resulting transcriptional modules of co-expressed genes. (D) GO analysis and KEGG analysis of genes whose expression was upregulated after BCP injury. (E) GO analysis and KEGG analysis of genes whose expression was downregulated after BCP injury. (F) GSEA of KEGG after BCP injury. *p* < 0.05 was significantly enriched.

After BCP formation, the genes of astrocytes also underwent significant changes (Figure 7(A)). Compared with the control group, the genes Prkacb, Gja1, Gnai2, Tuba1a, Tubb4a, Cldn11, etc. were significantly upregulated in the BCP group, while Plcb1, Plcb4, Itpr2, Itpr1, Rhh1, Adcy9, Grin2b, Grin2c, Gria1, etc. were downregulated (*p* < 0.05, Figure 7(B)). Monocle pseudo-time analysis further revealed the changes in gene expression modules after BCP (Figure 7(C)). The GO and KEGG analysis of differentially expressed genes showed that BCP could cause significant enrichment of spinal astrocytes in membrane remodelling, metabolic reprogramming, neurodegenerative pathways, and lysosome/apoptosis enhancement pathways, suggesting that astrocytes could drive neural inflammation and injury, forming a vicious pain cycle (Figure 7(D)). The GO and KEGG analysis of differentially expressed genes showed that the synaptic regulatory function of astrocytes was significantly impaired (Figure 7(E)). This includes glutamatergic synaptic function and postsynaptic membrane; ion channel and calcium signalling pathway functions were inhibited. The downregulation of cell connection and plasma membrane-related genes suggested that the integrity of the astrocyte foot structure might be disrupted under the BCP model, further affecting the BSCB function and the stability of the synaptic microenvironment. Lastly, the analysis of GSEA indicates that in the BCP model, astrocytes in the spinal dorsal horn show significant positive enrichment in oxidative phosphorylation, neurodegenerative diseases, and Parkinson’s disease (Figure 7(F)). This suggests that BCP induces a functional remodelling similar to that in neurodegenerative diseases and Parkinson’s, mainly by inducing mitochondrial stress and dysfunction, causing astrocytes to enter a ‘reactive’ state and shift towards the A1 pro-inflammatory state.

In the BCP model, the three major components of BSCB - endothelial cells, pericytes and astrocytes - all exhibited significant functional disorders. The disruption of endothelial cell connections and inflammatory activation, the loss of vascular support function of pericytes, and the pro-inflammatory and ­terminal foot structure damage of astrocytes jointly led to the impairment of the integrity of BSCB. This disruption of barrier function not only provided conditions for the infiltration of immune cells and inflammatory factors, but also might further promote central sensitization and the chronicity of pain by altering the spinal microenvironment.

### 3.6. Molecular characteristics of neurons after BCP

In the pathological process of BCP, the plasticity changes of spinal cord neurons are an important mechanism leading to central sensitization and pain persistence. Studies have shown that BCP not only enhances the activity of excitatory neurons but also weakens the function of inhibitory neurons. This disruption of the excitation-inhibition balance jointly promotes the abnormal activation of pain signaling pathways [19,20].

**Figure 8.**
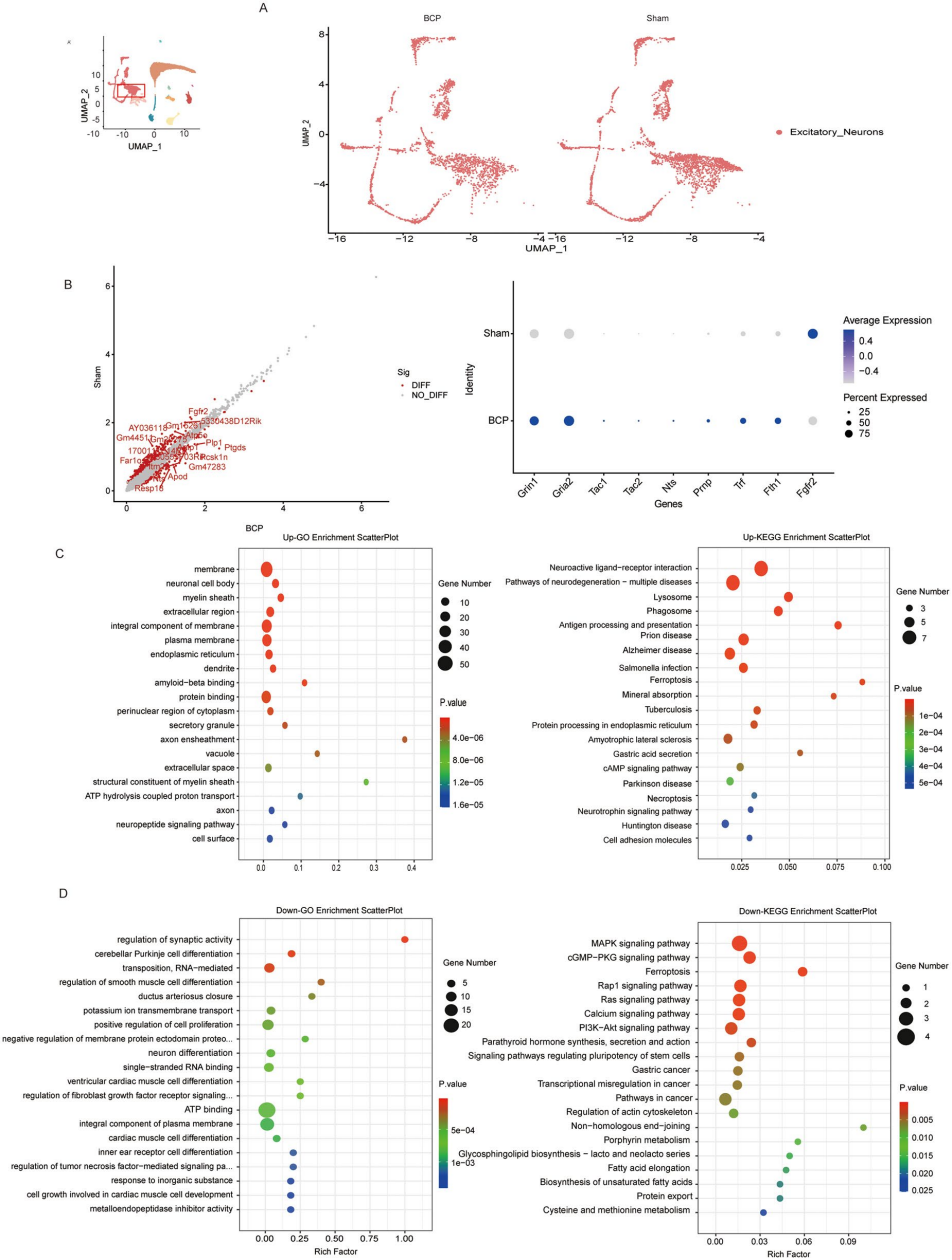
The characteristic changes in molecular markers of excitatory neurons by BCP. (A) UMAP visualization of excitatory neurons in the sham and BCP groups. (B) Volcano plot of differentially expressed genes in endothelial cells between the control group and the BCP group, as well as dot plots showing the expression levels and frequencies of some of the differentially expressed genes. (C) GO analysis and KEGG analysis of genes whose expression was upregulated after BCP injury. (D) GO analysis and KEGG analysis of genes whose expression was downregulated after BCP injury. *p* < 0.05 was significantly enriched.

Comparing the control group and BCP group, the excitatory neurons in different genes (Figure 8(A)). We found Grin1, Gria2, Tac1, Tac2, Nts, CD81, Prnp, agency, Fth1 raised such as gene, Fgfr2, Atp5o, P2ry12, Scn9a, Ctnna3 and others were downregulated (*p* < 0.05, Figure 8(B)). GO and KEGG analyses of the upregulated differentially expressed genes showed that BCP could cause significant enrichment of excitatory neurons in pathways such as neuronal structural damage, neuroinflammation and immune activation, and abnormal synaptic signal transmission (Figure 8(C)). For instance, GO analysis shows that genes related to neuropeptide signalling pathways and dendrite/axon structures are upregulated, supporting neuronal overexcitation. The genes related to extracellular regions and myelin structures are upregulated, reflecting immune cell infiltration and myelin damage. In the corresponding KEGG analysis, pathways such as antigen presentation and phagosomes were enriched, indicating that excitatory neuron-mediated neuroinflammation was activated. The interaction of neuroactive ligands and receptors and the significant enrichment of cAMP signals suggest that excitatory neurons enhance pain signal transduction. GO and KEGG analyses of differentially down-regulated genes also indicated abnormal regulation of synaptic activity in excitatory neurons, accompanied by dysregulation of MAPK, calcium signaling, PI3K-Akt, and ferroptosis signalling pathways (Figure 8(D)).

**Figure 9.**
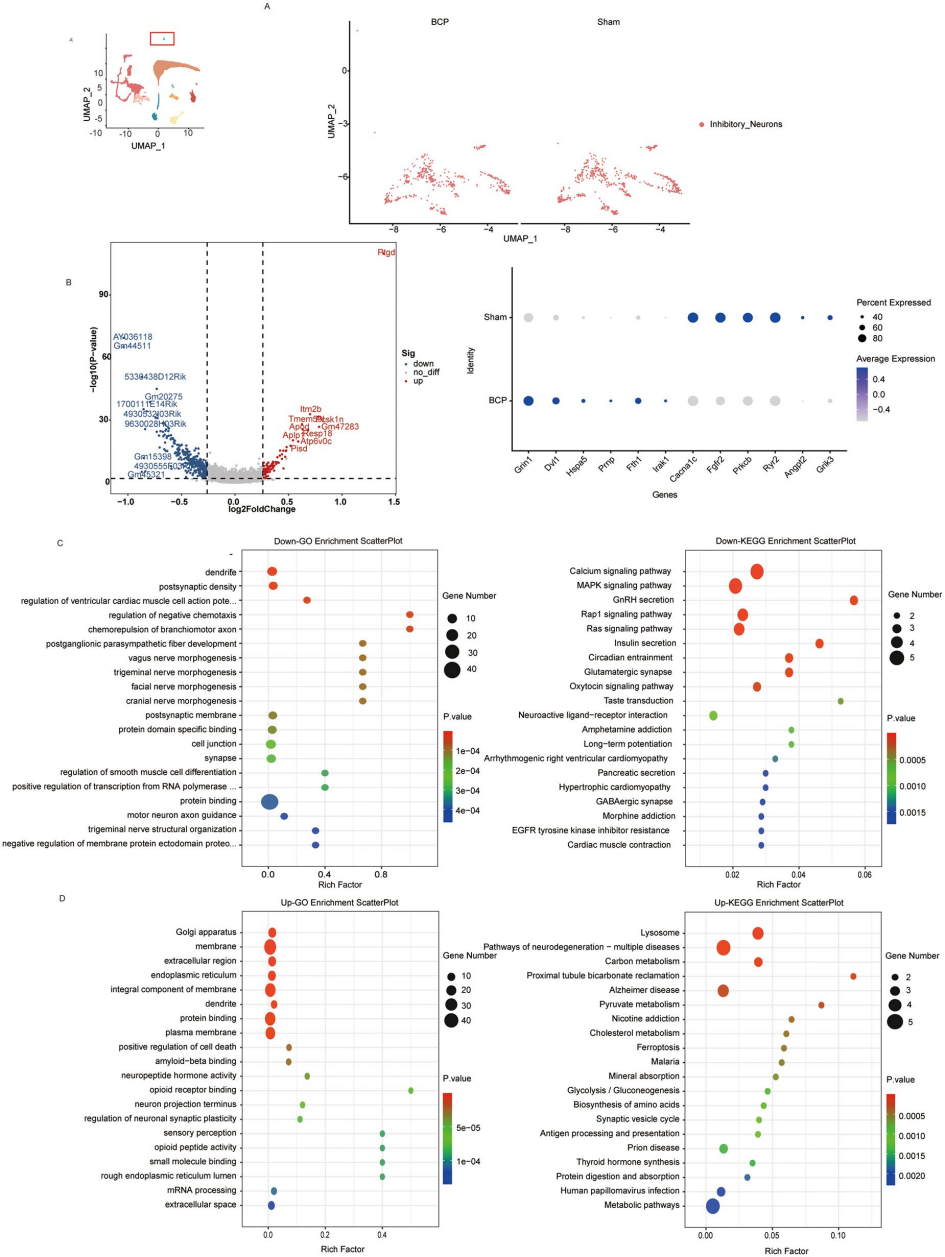
The characteristic changes in molecular markers of inhibitory neurons by BCP. (A) UMAP visualization of inhibitory neurons in the sham and BCP groups. (B) Volcano plot of differentially expressed genes in inhibitory neurons between the control group and the BCP group, as well as dot plots showing the expression levels and frequencies of some of the differentially expressed genes. (C) GO analysis and KEGG analysis of genes whose expression was downregulated after BCP injury. (D) GO analysis and KEGG analysis of genes whose expression was upregulated after BCP injury. *p* < 0.05 was significantly enriched.

The gene expression profiles of inhibitory neurons in the control group and the BCP group were compared (Figure 9(A)). We found that genes such as Grin1, Dvl1, Hspa5, Prnp, Fth1, Irak1 were upregulated, while genes such as Cacna1c, Fgfr2, Prkcb, Ryr2, Angpt2, Grik3 were downregulated (*p* < 0.05, Figure 9(B)). The GO and KEGG analyses of the upregulated differentially expressed genes showed that bone cancer pain could cause enrichment of inhibitory neurons in pathways such as neuronal structure and function remodelling, pro-death signalling, and immune inflammatory response (Figure 9(C)). For example, in the GO analysis, positive regulation of cell death and ferroptosis were enriched, suggesting that it may exacerbate neuronal damage through pathways such as ferroptosis. Synaptic plasticity regulation and synaptic vesicle cycling were significantly enriched, indicating that bone cancer pain may cause enhanced pain signal transmission by altering synaptic transmission efficiency and neurotransmitter release mechanisms. Dendrite structure and neuronal projection terminal-related genes were upregulated, suggesting that neuronal morphology undergoes adaptive changes and may participate in the long-term strengthening of the pain pathway. The antigen presentation pathway was enriched in KEGG, suggesting the role of neural-immune interaction in bone cancer pain. The GO and KEGG analyses of the downregulated differentially expressed genes indicated that in the BCP model, inhibitory neurons had significant functional and structural damage and dysregulation of key signalling pathways (Figure 9(D)). For example, in the GO, synaptic-related structures and neuronal morphogenesis were enriched, and in KEGG, GABAergic synapse and Glutamatergic synapse were enriched, both suggesting that the synaptic transmission function (especially GABAergic inhibition) and the integrity of synaptic structure of inhibitory neurons were disrupted, possibly leading to the failure of pain inhibition function. The key signalling pathways such as calcium signalling, MAPK/Ras/Rap1 signalling, and neuroactive ligand–receptor interaction were dysregulated, suggesting enhanced pain-related signal transduction and weakened inhibitory regulatory mechanisms.

### 3.7. Intercellular interactions of spine cord caused by BCP

**Figure 10.**
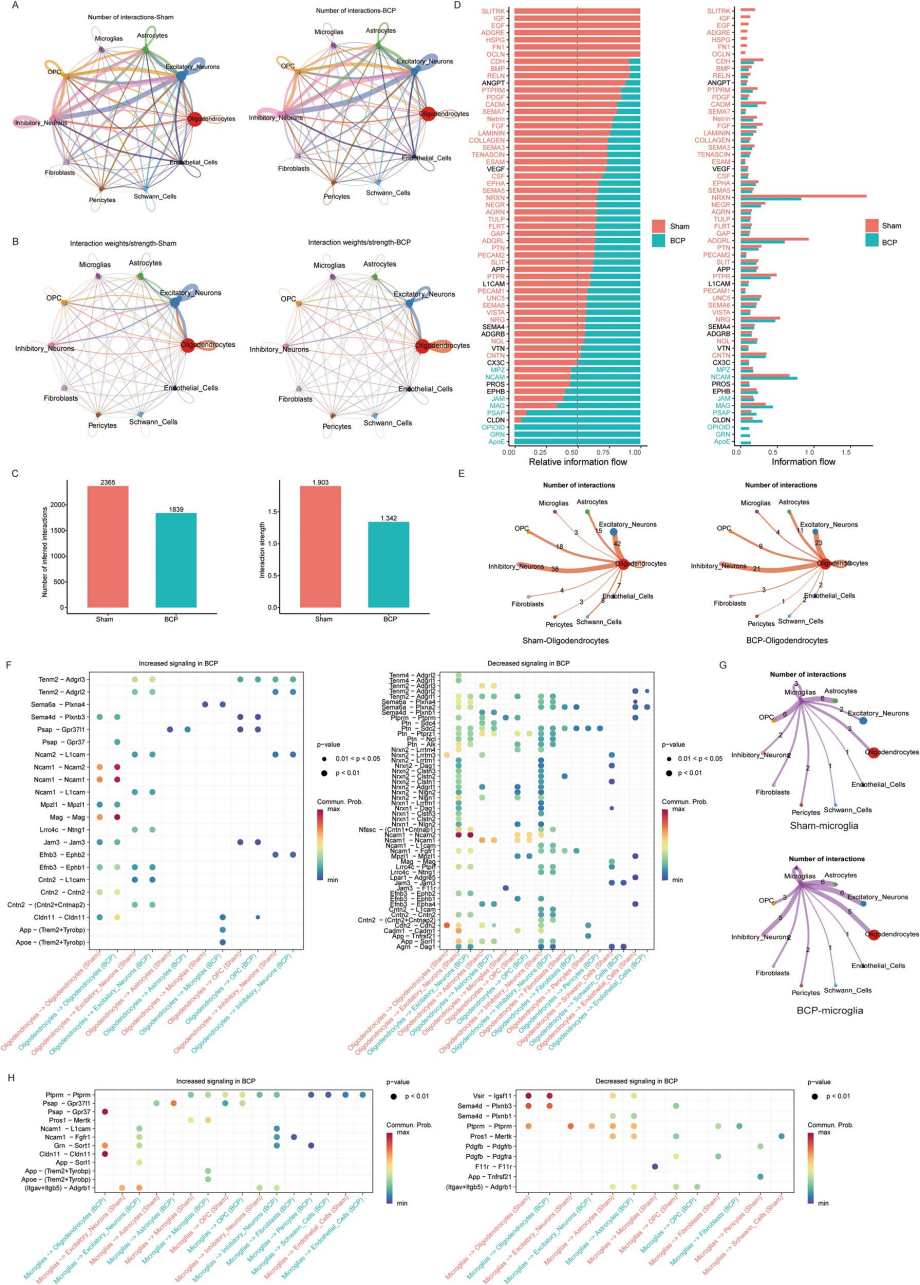
Intercellular interactions of spine cord caused by BCP. (A–C) Circo plot showing predicted (by CellChat) cell-cell communication among all 10 spine cell types. Node number and weights represent the number of significant ligand–receptor pairs between two cell types. (D) The relationship of information flow in the sham and BCP groups is shown in the right panel. (E) The number of the inferred oligodendrocytes-other cells signalling network. (F) Ligand–receptor pairs increased and decreased between oligodendrocyte sand other cells in the sham and BCP groups. Dot colour and size represent the calculated communication probability with P values computed using a one-sided permutation test. (G) The number of the inferred microglia-other cells signaling network. (H) Ligand–receptor pairs increased and decreased between microglia and other cells in the sham and BCP groups. Dot colour and size represent the calculated communication probability with P values computed using a one-sided permutation test. *p* < 0.05 was considered significantly enriched.

The spinal cord is composed of various cells, which interact with each other to ensure homeostasis. Therefore, we used CellChat to explain the complex intercellular communication network after BCP injury (Figure 10(A–B)). Corresponding to BCP, the number of cell type interactions in L2-L4 spinal cord tissue decreased from 2365 to 1839, and the interaction strength dropped from 1.903 to 1.342 (Figure 10(C–D)). Further, the cell communication networks between oligodendrocytes and other cells, as well as the increase and decrease of ligand–receptor pairs between cells, were extracted. Compared with Sham group, the number of interactions between oligodendrocytes and microglia in the BCP model was increased. On the contrary, the interactions with inhibitory neurons, excitatory neurons, OPCs, etc. were weakened (Figure 10(E)). After BCP injury, the interaction between oligodendrocytes and microglia through the App, Apoe and Cldn11 pathways increased, while the Ptprm pathways have a decrease in signal transmission. The Mag and Cldn11signaling pathways are increased between oligodendrocytes; meanwhile, the Cdh2 and Nrxn2-Lrrtm3 signalling pathways are weakened. Moreover, the Cadm1, Cdn2, Nrxn1, Nrxn2, and Ptprm pathways reduced the signal transmission between oligodendrocytes and excitatory neurons. In these pathways, Cadm1, Cdn2, Nrxn1, and Nrxn2 also reduced the interaction signal between oligodendrocytes and inhibitory neurons. Ptprm and Mpzl1 pathways have a decrease in signal transmission between oligodendrocytes and endothelial cells. Nrxn-Lrrtm signalling pathways are weakened between oligodendrocytes and astrocytes, OPC, Schwann cells (Figure 10(F)). In summary, under the BCP model, the communication network between oligodendrocytes and other cell types (including themselves, neurons, astrocytes, microglia, etc.) undergoes significant reorganization.

Further, the cell communication networks between microglia and other cells, as well as the increase and decrease of ligand–receptor pairs between cells, were extracted. Corresponding to BCP, the number of cell type interactions is increased between microglia and excitatory neurons as well as inhibitory neurons (Figures 10(G)). We found that after BCP injury, microglia and excitatory neurons interacted more through the App, Grn, and Ncam1 pathways. In these pathways, microglia also interacted with inhibitory neurons through the Grn and Ncam1 pathways. Meanwhile, the Apoe/App-Trem2 + Tyrobp signaling pathways are increased between microglias. The Pasp-GPR37l1 signaling pathways is enhanced between microglia and astrocyte/OPC. The Grn -Sort1 and Ptprm-Ptprm signalling pathways is enhanced between microglia and pericytes. Ptprm pathways is enhanced between microglia and Schwann cells. The down-regulated signalling pathways mainly include Sema4d, Pros1, Pdgfb, F11r, Tnfrsf21. We found that signalling of Sema4d, Pros1 and Pdgfb from microglias to OPC were decreased. F11r was decreased between microglias. Pdgfb andTnfrsf21 pathway activity were downregulated from microglias to pericytes (Figure 10(H)).

## 4. Discussion

BCP has a unique and complex pathophysiological mechanism, and the specific molecular mechanisms underlying it still need to be further explored. In this study, bone cancer pain model induced by Lewis lung cancer cells in male mice was established, and the corresponding cell atlas of the spinal cord segments was analyzed through single-cell sequencing methods. We determined 10 cell types based on different marker genes. In addition, we further detected the changes in gene expression profiles of oligodendrocytes, microglia, astrocytes constituting the blood-spinal cord barrier, pericytes and endothelial cells, as well as excitatory and inhibitory neurons between the Sham group and the BCP group. Finally, we analysed the changes in cell communication and molecular characteristics among various cells in the BCP model.

Oligodendrocytes are the main cells that produce myelin in the central nervous system and are crucial for neural signal transmission. Studies have shown that oligodendrocytes are involved in the development of various pain models, such as neuropathic pain, cancer pain, diabetic neuropathy pain, etc [21,22]. In the BCP model, the differentiation defect of oligodendrocytes and the loss of myelin are directly related to pain behavior [23]. In the pain-induced diabetic neuropathy rat model, it was found that the number of oligodendrocytes in the dorsal horn of the spinal cord significantly increased, meanwhile the morphological plasticity of oligodendrocytes (such as imbalance of proliferation and apoptosis) is a key mechanism of diabetes-related pain [24]. Our single-cell sequencing results also suggest that in the BCP model, the proportion of oligodendrocytes in L2-L4 spinal cord tissue cells is significantly upregulated. GO and KEGG analysis indicate that there are obvious lipid metabolism abnormalities in oligodendrocytes, which can cause damage to the structure and function of myelin. For example, the differential gene analysis of oligodendrocytes suggests that the expression of genes such as Prnp (prion protein) and Apoe (apolipoprotein E) is upregulated. These genes have obvious neurotoxicity and can cause damage to the structure of myelin [25]. Scd3, B3galt2, and Hexb expression is significantly downregulated. These genes can participate in lipid/sphingolipid disorders and further cause damage to the structure of myelin. In addition, studies have shown that oligodendrocytes can also participate in the occurrence and development of pain by regulating inflammation and apoptosis [26]. The enrichment analysis of single-cell sequencing in this study also suggests that oligodendrocytes have apoptosis. Gu’s research has discovered that the LCN2 protein released by microglia induces ferroptosis in oligodendrocytes through specific signaling pathways, thereby promoting white matter damage [27]. In multiple sclerosis models, neuroinflammation disrupts the intrinsic protective mechanisms of oligodendrocytes, making them more prone to ferroptosis during the process of maturation and differentiation, leading to continuous loss of myelin-forming cells [28]. This study also found that BCP also causes ferroptosis in oligodendrocytes, but the specific mechanism requires further investigation to provide new targets for the treatment of BCP. Boscia F’s research also confirmed that calcium signal disorder can lead to the maturation disorder of oligodendrocytes [29]. The single-cell sequencing results of this study also indicate that in the BCP model, oligodendrocytes have significantly downregulated calcium channel activity, transport, and homeostasis. Currently, the specific mechanism and process by which oligodendrocytes still need further research in the BCP.

The spinal microglia, as the main immune effector cells of the central nervous system, play a complex and crucial role in the BCP. Studies have shown that in BCP, spinal microglia polarize towards the pro-inflammatory M1 phenotype (high expression of IL-1β), while the anti-inflammatory M2 phenotype (expressing IL-10) is suppressed [30]. Moreover, the P2X7 receptor-ATP-p38 MAPK-IL-18 signalling axis can enhance synaptic transmission of nociceptive neurons in the spinal dorsal horn and promote central sensitization [31–33]. The results of this study also suggest that BCP causes an increase in the number of microglia and significant changes in the gene expression profile of microglia. GO analysis shows that the immune and inflammatory signalling pathways of glial cells are activated, such as phagocytosis, lysosomal degradation, innate immune response, apoptosis (Apoptosis) and autophagy (Figure 4). Based on the characterization of microglial subpopulations by cell heterogeneity. We reclustered the microglia. Compared with the Sham group, the proportion of cluster 0 in the BCP group was increased. Further GSEA analysis also indicated that cluster 0 is a highly activated microglial cell subset with a clear pro-inflammatory phenotype in the BCP model, and is likely to be the key effector cell driving neuroinflammation and pain maintenance. The results of the differential gene expression analysis of cluster 0 indicated that Cd14b expression increased after BCP modelling, and Cd14 was closely related to pathways such as MAPK signalling pathway, NF-kappa B signalling pathway, and toll-like receptor signalling pathway. Thus, it can be speculated that after BCP modelling, microglia may promote their transformation to the pro-inflammatory phenotype by upregulating Cd14. Lan LS et al.’s study also confirmed that in the rat bone cancer pain model, the expression of CD14 in pro-inflammatory microglia cells was upregulated [34].

The BSCB is composed of endothelial cells and the underlying basal layer, pericytes, and astrocyte foot processes. Its function is similar to that of the blood–brain barrier [35]. Current literature indicates that the disruption of the BSCB in BCP models may be related to the release of inflammatory factors, oxidative stress, matrix degradation and direct tumour invasion [36,37]. The tight junction proteins between endothelial cells (such as Claudin 5, Occludin, ZO-1, etc.) are of vital importance for maintaining the integrity of the barrier [38]. In the BCP model, Evans blue dye extravasation increased (indicating an increase in barrier permeability), transmission electron microscopy showed structural abnormalities, and western blot detected decreased expression of tight junction proteins (such as ZO-1 and claudin-5) [36]. The GO and KEGG analysis of spinal endothelial cells in this study also suggested that the functional and integrity of endothelial cells were significantly dysregulated, such as the analysis of GO indicating enhanced lipid transmembrane transport, suggesting increased barrier permeability; the analysis of KEGG also indicated the downregulation of adhesion connections and Wnt pathways, meaning the disintegration of tight junction proteins, accelerating barrier leakage. In addition, the NOD inflammatory pathway of endothelial cells was also significantly activated. In conclusion, spinal endothelial cells mainly participate in the occurrence and development of pain through pro-inflammatory effects and the disruption of the integrity of the BSCB, increasing the permeability of the BSCB.

This study also analysed the change of pericytes under the BCP model. The results suggest that compared with the Sham group, the number of pericytes in the BCP group is significantly reduced. At the same time, the GO and KEGG analysis of the differential genes also indicate that the degeneration of vascular functions related to pericytes, such as angiogenesis and migration-related pathways, is significantly inhibited, suggesting the destruction of BSCB integrity. The activation of endoplasmic reticulum stress and apoptosis pathways may be the main mechanism for the significant reduction in the number of pericytes in the BCP model. In the spinal cord injury (SCI) animal model, it was found that inhibiting endoplasmic reticulum stress and reducing pericyte apoptosis can protect the integrity of BSCB and promote axon growth [39]. In ALS patients and neuropathic pain models, the absence of pericytes (such as a reduction in PDGFR-β+ and CD13+ cells) can lead to endothelial cell damage and downregulation of tight junction protein expression (such as claudin-5, ZO-1), causing BSCB leakage and IgG leakage [40,41]. However, the specific molecular mechanism needs to be further explored.

Most literature indicates that spinal astrocytes mainly participate in the occurrence and development of BCP by releasing various inflammatory factors, chemokines, and interacting with neurons [42,43]. The results of this study also show that after BCP injury, spinal astrocytes exhibit immune activation and upregulation of neuroinflammatory pathways. It is worth noting that in the Lewis lung cancer-induced male mouse bone cancer pain model, the number of astrocytes in the spinal dorsal horn was significantly reduced. This is not contradictory to the functional activation state reflected by the upregulation of GFAP expression observed in previous studies. Both may jointly constitute the complex response pattern of astrocytes in pain. Therefore, we further conducted GSEA analysis, and the results showed that in the BCP model, astrocytes showed significant positive enrichment in the oxidative phosphorylation pathway as well as in neurodegenerative diseases and Parkinson’s disease. This further suggests that BCP may induce mitochondrial stress and dysfunction, promoting the transformation of astrocytes to the A1 pro-inflammatory state, similar to that in Parkinson’s and Alzheimer’s diseases, indicating that astrocytes lose their neuro-supportive function and instead acquire the ability to promote neuronal damage/synaptic abnormal remodelling. Considering the GSEA results, we speculate that the potential mechanism for the reduction in the number of astrocytes may include the following aspects: Firstly, after the disruption of the blood-spinal cord barrier, tumour-related factors and inflammatory mediators in the peripheral circulation can directly invade the spinal dorsal horn microenvironment, activating apoptotic-related signalling pathways and inducing astrocytes to undergo apoptosis or pyroptosis, thereby causing a decrease in cell numbers; Secondly, the integrity of the blood-spinal cord barrier is crucial for maintaining the local microenvironment stability of the spinal cord. Barrier disruption may lead to the loss of neurotrophic factors in the spinal cord tissue fluid, weakening the support effect on the survival and proliferation of astrocytes; Thirdly, astrocytes may cause mitochondrial dysfunction to maintain high metabolism for toxin clearance or synthesis of inflammatory mediators. This metabolic failure is a common feature of degenerative diseases, suggesting that the glial cells in the cancer pain model may be in an ‘exhaustive’ maladaptive state. In late Alzheimer’s disease, chronic inflammation and pathological protein deposition continuously damage the blood–brain barrier and astrocytes. Astrocytes gradually shift from an early reactive state to a functionally deteriorated state, experiencing nutrient support failure and ion homeostasis imbalance, ultimately leading to cell death and a decrease in cell numbers [44]. However, there are still limitations in our demonstration of the causal relationship between the reduction in the number of astrocytes and the change in the functional state of astrocytes or the disruption of the blood-spinal cord barrier, and further verification is still needed.

Spinal cord neuron dysfunction is also one of the important mechanisms of BCP. The results of this study identified that the spinal cord neurons mainly include inhibitory neurons and excitatory neurons. Among them, excitatory neurons include glutamate-expressing excitatory neurons, excitatory synapses, and sensory-specific neurons in the dorsal root ganglion (DRG). The core mechanism of excitatory neurons in BCP includes: synaptic structure remodelling (such as dendritic spine hyperplasia), upregulation of excitatory receptors (GluR1/GluN1), enhancement of glutamate transmission, and increased spontaneous neuronal firing [45,46]. These changes are precisely regulated by multiple signalling pathways (such as GPR30, Rac1/PAK1, Slit2/Robo1, VEGFA, etc.), jointly promoting central sensitization and the persistence of pain [47,48]. In 2024, the research conducted by Xu C et al. further clarified that epigenetic modifications in DRG play a crucial role in the occurrence and development of BCP, and revealed a novel epigenetic mechanism: during the BCP process in rodents, the regulation of histone modifications can enhance the binding ability of transcription factor Sp1 to the promoter of the Gpr160 gene, thereby promoting the transcription of the Gpr160 gene and the expression of GPR160 in nociceptive DRG neurons after tumour infiltration, and facilitating the occurrence and development of cancer pain [49]. Thus, in the context of neurons and transcriptional regulation, the regulation of epigenetic processes is becoming increasingly important in BCP, and different epigenetic processes play different roles at various stages of pain processing [3]. This study suggests that in the BCP state, excitatory neurons exhibit significant molecular characteristic changes. The expression of glutamate receptor-related genes such as Grin1 and Gria2 is upregulated, indicating enhanced function of NMDA receptors and AMPA receptors, which will directly lead to an increase in the efficiency of glutamate synaptic transmission. This enhancement of synaptic efficacy may be the key molecular basis of central sensitization. At the same time, the expression of various excitatory neuropeptides such as Tac1, Tac2, and Nts increases, further amplifying the transmission of pain signals. The GO and KEGG analysis of the differential genes of excitatory neurons also suggest that the neuropeptide signalling pathway and the corresponding neural activity ligand–receptor interactions are significantly activated, including pain-related neurotransmitters regulation (such as substance P) and pain signal transmission (GPCR, ion channels). However, the specific molecular mechanism needs further exploration.

Contrary to the changes in excitatory neurons, inhibitory neurons exhibit distinct functional inhibitory characteristics in the BCP model [50]. The study by Ding Z et al. demonstrated that BCP can trigger ferroptosis in spinal GABAergic intermediate neurons, further leading to the loss of GABAergic neurons and thereby weakening the inhibitory regulation of the spinal cord on pain signals, resulting in the amplification of pain signals [9]. Moreover, the decline in the function of inhibitory neurons is not only manifested in their own death or changes in excitability, but also leads to the disruption of the balance between excitatory (glutamatergic) and inhibitory (GABAergic) transmission at the spinal dorsal horn level. For example, in the SCI model, the reduction in the activity of inhibitory neurons leads to excessive activation of excitatory neurons, thereby disrupting the balance between excitability and inhibition [51]. This imbalance not only leads to the aggravation of pain but may also trigger the degeneration of motor neurons [52]. The single-cell sequencing results of this study identified inhibitory neurons mainly as γ-aminobutyric acid (GABA)ergic neurons (GABAergic inhibitory neurons). Compared with the Sham group, the number of inhibitory neurons in the BCP group was significantly reduced, and the results of GO and KEGG analysis of the differential genes of inhibitory neurons also indicated an imbalance between GABAergic synapses and glutamatergic synapses, accompanied by positive regulation of cell death and ferroptosis in GABAergic inhibitory neurons. Through in-depth research on these mechanisms, new ideas and targets for the treatment of related diseases can be provided.

BCP injury can lead to an increase in the percentage of oligodendrocytes to microglia in the spinal cord, and at the same time, the proportion of excitatory neurons and inhibitory neurons related to pain signal transmission is significantly reduced. Finally, we used CellChat to mainly analyse the interaction relationships between oligodendrocytes and microglia, as well as between oligodendrocytes and excitatory neurons and inhibitory neurons, respectively. We found that after BCP injury, the enhanced ligand-receptor pairs between oligodendrocytes and microglia include APP-Trem2 and Apoe-Trem2; while the Ptprm and Jam pathways were weaken between oligodendrocytes and microglia; and the Cadm1, Cdn2, Nrxn1, and Nrxn2 signals were reduced between oligodendrocytes and excitatory neurons as well as inhibitory neurons. In the AD model, it is mentioned that oligodendrocytes participate in the generation of APP, which can cause dysfunction in myelin maintenance; at the same time, APP, as a core pathological protein, can activate microglia and release a large amount of inflammatory factors to accelerate neuronal damage. This phenotypic transformation is closely related to abnormal receptor signaling pathways such as TREM2[54]. Thus, oligodendrocytes can produce and release App and its fragments, which is equivalent to directly sending ‘danger signals’ to microglia, further recruiting and activating them, thereby causing the occurrence and development of BCP. However, further experiments are still needed for verification. We analysed the interaction relationship between microglia and excitatory neurons as well as inhibitory neurons. We found that BCP significantly enhanced the communication between microglia and excitatory neurons, as well as inhibitory neurons. The involved ligand-receptor pairs included App-Sort1, Grn-Sort1, and Ncam1-Fgfr1. Studies have shown that App, Grn, and Sort1 are all key regulators of inflammation [53–55]. And Deng M et al.’s research found that Grn increased the expression of pro-inflammatory cytokines and chemokines in TREM2 macrophages, and regulating the Grn-Sort1 signalling pathway might be one of the targets for treating acne [56]. Our research also found that in the BCP model, the Grn-Sort1 signalling between microglia and excitatory neurons was enhanced, suggesting a possible inflammatory-pain mechanism between the two, but a more in-depth mechanism study still needs to be further explored.

Actually, this study has certain limitations: Firstly, single-cell RNA sequencing was conducted only on BCP model induced by Lewis lung cancer cells in male mice. The pathological differences in bone metastasis of other tumour types may affect the generalizability of the results; Secondly, no female samples were included, and the influence of gender differences on pain and immunity was not investigated; Finally, although the sample size was estimated and in accordance with the ‘3 R principles’ of animal ethics, it may still not be sufficient to reveal all subtle differences. In the future, multiple tumour type BCP models will be established, including both male and female animals, and combined with multi-omics and clinical data, to provide a more comprehensive basis for precise treatment. Based on the findings of this study, Sgk1 is expressed at higher levels in microglia and the lysosomal pathway is enriched. We plan to conduct systematic mechanism research around ‘SGK1 drives bone cancer pain by disrupting the lysosomal function of microglia’, including: (1) Phenotypic verification: Confirm Sgk1 expression and activation through RNAscope/immunofluorescence, and correlate autophagy flow, lysosomal function with pain behavior; (2) *In vivo* functional verification: Observe the effects of drug inhibition or gene knockout on BCP and lysosomal function, and combine with immunoelectron microscopy to observe ultrastructure; (3) *In vitro* mechanism analysis: In microglia stimulated by bone cancer cell-conditioned medium, use mRFP-GFP LC3 dual-labelled adenovirus, lysosomal live cell staining and TFEB nuclear translocation analysis to determine whether SGK1 damages lysosomal biosynthesis and function by inhibiting TFEB.

## 5. Conclusion

In summary, by focusing on individual cells, we have developed a transcriptom atlas of the spinal cord tissue after BCP. At the cellular level, understand the changes in the gene expression characteristics of individual cells in spinal cord tissue after BCP injury. In addition, we also investigated the interactions between oligodendrocytes and microglia, as well as between microglia and spinal cord neurons after BCP injury. Our data will lay the foundation for a deeper understanding of the molecular mechanisms underlying the occurrence and development of BCP and help identify new intervention signalling pathways.

## Data Availability

The data that support the findings of this study are available from the corresponding author upon reasonable request.
